# Osmolality of Commercially Available Oral Rehydration Solutions: Impact of Brand, Storage Time, and Temperature

**DOI:** 10.3390/nu11071485

**Published:** 2019-06-29

**Authors:** Kurt J. Sollanek, Robert W. Kenefick, Samuel N. Cheuvront

**Affiliations:** 1 Department of Kinesiology, Sonoma State University, Rohnert Park, CA 94928, USA; 2 Sports Science Consulting, LLC, Hopkinton, MA 01748, USA; 3 Sports Science Synergy, LLC, Franklin, MA 02038, USA

**Keywords:** oral rehydration therapy, dehydration, rehydration, euhydration, electrolytes

## Abstract

Oral rehydration solutions (ORS) are specifically formulated with an osmolality to optimize fluid absorption. However, it is unclear how many ORS products comply with current World Health Organization (WHO) osmolality guidelines and the osmotic shelf-life stability is not known. Therefore, the purpose of this investigation was to examine the within and between ORS product osmolality variation in both pre-mixed and reconstituted powders. Additionally, the osmotic stability was examined over time. The osmolality of five different pre-mixed solutions and six powdered ORS products were measured. Pre-mixed solutions were stored at room temperatures and elevated temperatures (31 °C) for two months to examine osmotic shelf stability. Results demonstrated that only one pre-mixed ORS product was in compliance with the current guidelines both before and after the prolonged storage. Five of the six powdered ORS products were in compliance with minimal inter-packet variation observed within the given formulations. This investigation demonstrates that many commercially available pre-mixed ORS products do not currently adhere to the WHO recommended osmolality guidelines. Additionally, due to the presence of particular sugars and possibly other ingredients, the shelf-life stability of osmolality for certain ORS products may be questioned. These findings should be carefully considered in the design of future ORS products.

## 1. Introduction

Dehydration stemming from cholera and non-cholera (e.g., viruses, etc.) sources is a major cause of hospitalization and even mortality in many parts of the developed and less-developed world [1,2]. Key advances were made in the 1960s with the identification of electrolyte losses in diarrhea and the subsequent improvements seen in patients following administration of solutions containing glucose and electrolyte for oral rehydration known as oral rehydration therapy (ORT) [3,4,5]. Although these oral rehydration solutions (ORS) were successful in treating dehydration, the physiological mechanisms involving fluid absorption were not fully established until many years later, with the identification of the sodium–glucose transporters and many other dynamic mechanisms related to net fluid absorption [6,7,8,9,10,11]. Since these initial findings, many variations of the ORS have been created in an attempt to optimize efficacy [12,13]. 

Initially, the World Health Organization (WHO) and the United Nations Children’s Fund (UNICEF) advocated for an ORS with specific amounts of electrolytes and a slightly hypertonic osmolality of 311 mmol/kg [5]. However, this solution lacked broad usage due to gastrointestinal upset, nausea, and concerns about hypernatremia [14]. Additionally, this solution did not appear to significantly reduce stool output [13]. Consequently, the WHO and UNICEF revised their recommended formulation advocating for an ORS with a reduced electrolyte content and a corresponding lower osmolality of 245 mmol/kg [15]. In addition, studies of luminal perfusion indicate that beverages should be hypotonic with an osmolality range of 200 to 260 mmol/kg, which has been shown to facilitate the greatest rate of net fluid absorption [16].

It is important to note that many commercial ORS formulations are available and are sold as either pre-mixed solutions or in powdered form. However, it is unclear how many commercially available ORS products meet the 245 mmol/kg (200–260 mmol/kg) recommendation since beverage osmolality is not required for nutrition labeling; hence, there is a need for independent verification of WHO ORS osmolality compliance. The shelf-life stability of beverage osmolality is also not known. However, the solid-state stability of ORS powder is ingredient and environment dependent [17]. For example, pre-mixed liquid ORS can be sent to underdeveloped areas that lack clean water [18] and subsequently expose these products to prolonged storage with high environmental temperatures [19]. These conditions could increase beverage osmolality due to temperature-dependent sugar hydrolysis reactions depending on the sugars and other product ingredients [20].

Based upon the aforementioned questions, the current investigation was designed to answer the following: 1) What is the baseline osmolality and variability within commercial pre-mixed ORS? 2) what is the baseline osmolality and variability within commercial powdered ORS after reconstitution? and 3) what is the impact of the storage time and temperature on the osmolality of pre-mixed ORS? 

## 2. Materials and Methods 

### 2.1. Drink and Drink Preparations

Commercially available ORS were obtained from online retailers and local stores. The current investigation examined four different pre-mixed ORS, one pre-mixed sports drink, and six powdered ORS. The following pre-mixed drinks were analyzed: Pedialyte^®^ Classic mixed fruit flavor (Abbott Laboratories, Columbus, OH, USA), Pediatric Oral Electrolyte Solution fruit flavor (Up & Up, Target Brand, Minneapolis, MN, USA), Speedlyte^®^ wild orange flavor (Einsof Biohealth, Dover, DE, USA), and enterade^®^AD ORS orange flavor (Entrinsic Health Solutions, Inc, Norwood, MA, USA). Gatorade^®^ orange flavor (Pepsico, Chicago, IL, USA) was tested as a representative sports drink, since these are commonly used [21] or recommended [22] in the treatment of diarrhea, despite no marketing claims for this use. Product ingredients are listed in Table A1 (Appendix A). Lastly, six ORS powders were assessed: Hydralyte™ orange flavor (Hydralyte LLC, Thomastown, Victoria, Australia), Ceralyte^®^ 70 lemon flavor (Cera Products Inc., Hilton Head Island, SC. USA), Dioralyte™ citrus flavor (Sanofi, Guildford, Surrey, UK), Dioralyte™ Relief raspberry flavor (Sanofi, Guildford, Surrey, UK), DripDrop^®^ lemon flavor (DripDrop Hydration PBC, Oakland, CA, USA), and TRIORAL Oral rehydration salts natural flavor (Trifecta Pharmaceuticals, Ft. Lauderdale, FL, USA). 

### 2.2. Study Overview

The current investigation was comprised of three separate studies. The first study tested the baseline osmolality and variation of pre-mixed ORS products. To accomplish the first study, three new bottles of each pre-mixed ORS were opened and the osmolality was immediately assessed. This allowed us to examine the variability that was present due to manufacturing (inter-bottle variability). Within each beverage product type, the products had the same expiration date.

The second study assessed the baseline osmolality and variation within carefully reconstituted ORS powders. All powdered ORS varieties were prepared with room temperature distilled water (Market Pantry, Target Brand, Minneapolis, MN, USA) according to the respective manufacturers’ instructions. Three separate packets of each ORS were individually prepared and sampled to assess inter-packet variability and all three packets had the same expiration date. The dry powder from each packet was carefully diluted with the appropriate volumes of fluid using a calibrated scale. To enhance uniformity during drink preparation, each combination of powder and water was set to mix on the same Cimarec^®^ magnetic stirring plate (Barnstead/Thermolyne, Dueuque, IA, USA) at an equivalent speed for precisely 5 min. Immediately thereafter, samples were pipetted into sampling cuvettes for osmolality assessment.

**Figure 1:**
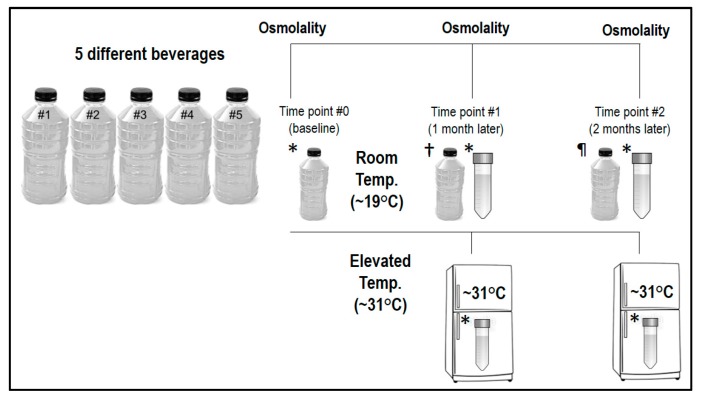
Schematic outlining the study methodologies for the third experiment; see text for further details. * The baseline bottle was aliquoted and subsequently stored for sampling at each time point. ^†,¶^ A new bottle of each test beverage was also sampled at each time point.

The third study examined the impact of storage time and temperature on beverage osmolality in the pre-mixed products. Details for this part of the investigation are depicted in Figure 1. At time point #0 (baseline), one new bottle of each pre-mixed ORS was opened and the osmolality was immediately assessed. The originally opened baseline bottle was then dispensed in 5 mL aliquots into 4 individual plastic centrifuge tubes (BIPPE, TS15; polypropylene). Subsequently, 2 tubes were stored at room temperature (~19 °C) and 2 tubes were stored at an elevated temperature (~31 °C) in a laboratory incubator. One tube from each beverage stored at room temperature and the elevated temperature was assessed 1 month later (time point #1) and 2 months later (time point #2) to determine the impact of storage time and temperature on osmolality measures. 

Additionally, at time points #1 and #2, a new pre-mixed bottle of each ORS was opened and assessed. These unopened bottles were stored concurrently with the tubes at room temperature during the ensuing months. Within each beverage type, all three bottles sampled had the same expiration date. Our rationale for measuring a freshly opened bottle of each beverage during the two different time points was to assess whether our method of storing beverages in centrifuge tubes qualitatively altered the beverages; hence, the fresh bottles served as an internal control. 

### 2.3. Osmometry

The main variable assessed in the current investigations was osmolality. Every beverage had at a minimum, osmometry performed in triplicate using a 250 μL sample on a freezing point depression device (Advanced^®^ Instruments 3250, Norwood, MA, USA). Importantly, recent work from our laboratory demonstrated that the use of larger sample volumes for osmolality measures gives greater uniformity in the values obtained and is less influenced by the sample composition [23,24]. When the triplicate intra-sample measures differed by ≤3 mmol/kg (~1%), the median value was used. If the triplicate intra-sample measures differed by >3 mmol/kg, two additional samples were measured and the median value was used [25]. The accuracy of the osmometer was confirmed at the start and completion of each testing session by assaying a known reference solution (Clinitrol™ 290, Advanced Instruments, Norwood, MA, USA) that listed an osmolality within the desired range of anticipated values. Reference solution measurements during all testing sessions were within the normal limits of the osmometer. Lastly, for all investigations, beverages were equilibrated to standard laboratory temperatures (~21 °C) prior to assessment. 

### 2.4. Water Content 

The percent of water in each pre-mixed beverage was ascertained using the thermogravimetric method based upon previously establish protocols in biological samples [26,27]. Briefly, 2 mL of each beverage was carefully weighed in an evaporation dish. Dishes were then heated over an open flame until visible moisture was gone (~2 min). Subsequently, dishes were placed in an electric oven (110 °C) for 10 mins for further desiccation. Dishes were cooled to room temperature before a final weight was obtained. The percentage of water content was calculated from the wet and dry weight. 

### 2.5. Statistics and Data Presentation

Standard statistics (e.g., mean, median, SD, percent coefficient of variation [%CV]) were calculated using Microsoft Excel^®^ 2016. All graphs were completed with the use of a computerized statistical software package (GraphPad Prism^®^ version 6 for Windows). Freshly sampled beverage osmolality values and samples subjected to storage were considered compliant and stable, respectively, if they fell within the 200 to 260 mmol/kg range [15,16]; those above or below the range were considered non-compliant. 

## 3. Results

### 3.1. Pre-Mixed ORS Variability

**Figure 2:**
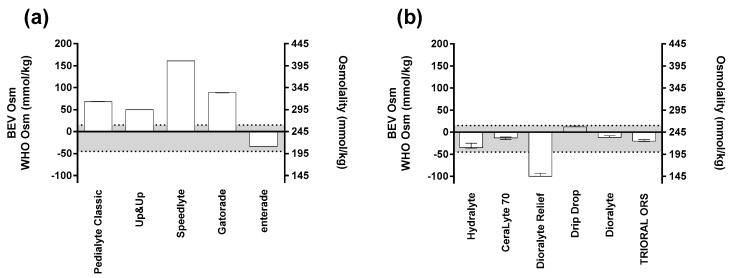
Beverage osmolality for: (**a**) pre-mixed solutions; and, (**b**) reconstituted powders. The left y-axis denotes the difference between beverage and WHO-UNICEF osmolality; the right y-axis denotes the absolute beverage osmolality. Each bar represents the median value for the three bottles tested. The variation plotted represents the range. The horizontal dotted lines denote the optimal range for beverage osmolality (200–260 mmol/kg; see text for details).

The results from the first investigation—examining the baseline osmolality values and variability of pre-mixed ORS—are shown in Figure 2a. Three bottles of each ORS were assessed to derive the variability that exists within a given formulation. Many of the beverages (enterade, Speedlyte, Pediatric Oral Electrolyte Solution) had no variation between bottles (0% coefficient of variation (CV)). Gatorade and Pedialyte had minimal variation with 0.17% and 0.18% CV, respectively. Lastly, the water content of the pre-mixed solutions was experimentally determined: Gatorade, 96%; Speedlyte, 97%; Pedialyte, 98%; Up & Up, 98%; and enterade, 99% water. 

Additionally, as shown in Figure 2a, a demarcation was placed between 200 and 260 mmol/kg to represent the osmolality range where the greatest rate of net fluid absorption occurred [16]. Of note, enterade was the only pre-mixed beverage that was within this standard at baseline. It is also important to note that many beverages at baseline were in excess of the original osmolality recommendation from the WHO (311 mmol/kg): Pedialyte Classic, ~313 mmol/kg; Gatorade, ~334 mmol/kg; and Speedlyte, ~406 mmol/kg.

### 3.2. Powdered ORS Variability

The results from the second investigation, examining the baseline osmolality values and variability of carefully reconstituted powdered ORS, are shown in Figure 2b. Three packets of powdered ORS were carefully reconstituted using distilled water according to each of the different manufacturer’s instructions. Immediately after the beverages were reconstituted, the osmolality was assessed to derive the variability that exists within and between the given formulations. Our results demonstrate that greater variation exists within the powdered forms compared to the pre-mixed solutions. The following is a list of the products sampled with their % CV, presented in order of their variation from lowest to highest: Drip Drop (0.45%), TRIORAL ORS (0.92%), Dioralyte original (0.99%), CeraLyte 70 (1.32%), Dioralyte Relief (2.74%), and Hydralyte (2.86%). In a similar fashion to the first investigation, Figure 2b denotes an optimal beverage osmolality range between 200 and 260 mmol/kg [16]. Of the six powdered ORS tested, only one (Dioralyte Relief; ~145 mmol/kg) had an osmolality outside of this range. 

### 3.3. Impact of Time and Temperature

**Figure 3:**
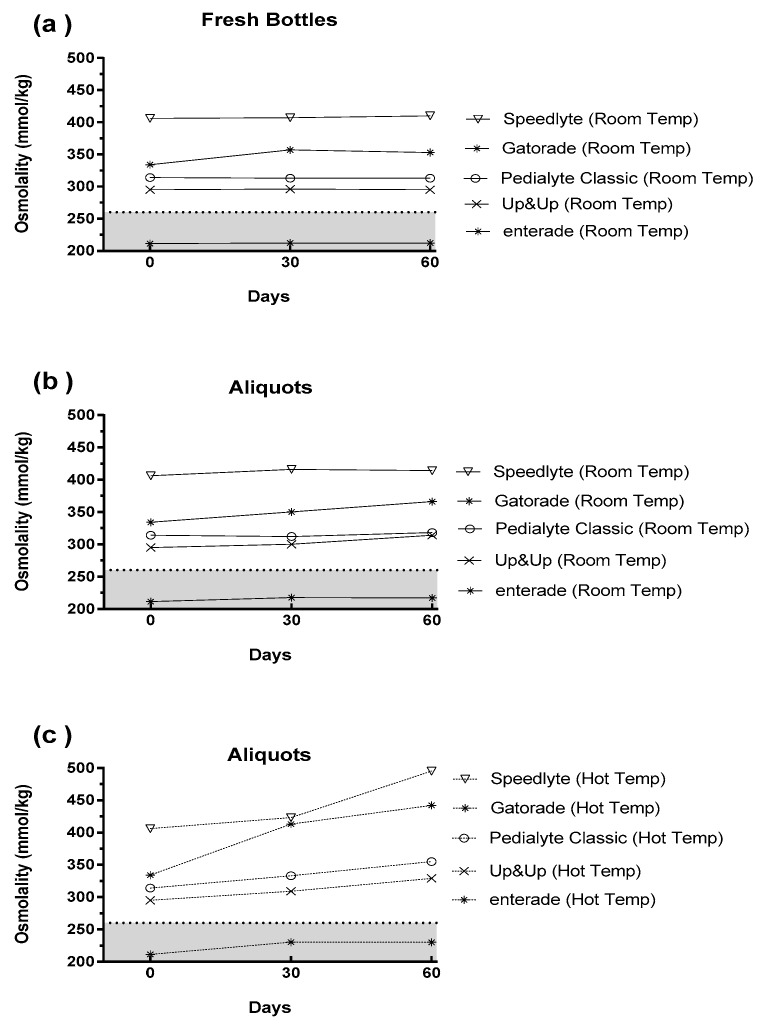
Impact of time and storage conditions on the osmolality of various pre-mixed oral rehydration solutions [ORS] for: (**a**) freshly opened bottles stored at room temperature; (**b**) beverage aliquots stored at room temperature; and, and (**c**) beverage aliquots stored at elevated temperatures. The shaded area denotes the optimal range for beverage osmolality (200–260 mmol/kg; see text for details).

The results from the third investigation, examining the impact of time and temperature on osmolality, are displayed in Figure 3. Figure 3a depicts the changes in pre-mixed ORS that occurred when the beverages were stored at room temperature in their original unopened package (i.e., “fresh bottles”). As expected, there was minimal change between baseline (0 days) and the 30- and 60-day samples. Figure 3b shows the data from the originally opened bottle, which was sampled at baseline and then pipetted into centrifuge tubes for subsequent storage at room temperature. These results closely mirror Figure 3a; thus, demonstrating that the storage of aliquots in centrifuge tubes did not inadvertently alter the osmolality. Lastly, Figure 3c shows the impact of storing beverages at elevated temperatures (~31 °C) [19]. These data demonstrate that many of the beverages had dramatic increases in osmolality during two months of storage at 31 °C. Of note, enterade demonstrated the lowest change from baseline (Δ 19 mmol/kg), while Speedlyte (Δ 89 mmol/kg) and Gatorade (Δ 108 mmol/kg) demonstrated the largest changes from baseline after 60 days in the hot temperatures.

Lastly, the same range for optimal beverage osmolality (200–260 mmol/kg) was placed in Figure 3 [16]. As previously shown, enterade was the only beverage that demonstrated osmolality values within this range at baseline and following room temperature storage. More importantly, enterade was still within this range even after 60 days at elevated temperatures.

## 4. Discussion

The present results demonstrate the following: 1) enterade was the only premixed ORS product that had baseline osmolality values between 200 and 260 mmol/kg and it stayed within this range after two months of storage at 31 °C; 2) some beverages, such as sports drinks and Speedlyte, had very high osmolality values at baseline and demonstrated robust increases during prolonged storage; and 3) little variation existed within premixed or powdered ORS products. 

The World Health Organization (WHO) and the United Nations Children’s Fund (UNICEF) currently recommend a reduced osmolality of 245 mmol/kg in their ORS formulations, which is a drop from the previous 311 mmol/kg recommendation [15]. The reductions in osmolality and electrolyte levels were a result of reported gastrointestinal upset, nausea, concerns about hypernatremia with the higher osmolality solution, and lack of reduced stool output [13,14]. It is important to note that research in healthy volunteers has demonstrated a range of hypotonic beverage osmolalities (200–260 mmol/kg) for optimal net intestinal fluid absorption [16] that includes the 245 mmol/kg recommended value in the treatment of diarrhea [14]. Since osmolality is not required on nutrition labeling, one aim of the current investigation was to independently verify which commercially available products are in compliance with these recommendations. Our results demonstrate high compliance with powdered ORS products since five of the six products analyzed fell within the desired range. However, of the five pre-mixed formulations tested, only one product (enterade) had an osmolality within the recommended range. These results call into question the overall ingredient composition within these products. 

A range of osmolality values for pre-mixed products was obtained at baseline in the current studies; ranging from a low of 211 mmol/kg (enterade) to a high of 406 mmol/kg (Speedlyte). Our results fall in-line with other limited data-sets demonstrating a wide range of osmolality values for ORS products [28]. Of note, many of the products tested had electrolyte values lower than those recommended by the WHO/UNICEF, and yet still had osmolality values in excess of the original osmolality recommendation of 311 mmol/kg. The main contributing factor to these elevated osmolality values appears to be the high sugar concentrations and the types of sugar used. For example, Gatorade was tested as a representative sports drink and the osmolality value was found to be ~334 mmol/kg, which aligns with other published values for Gatorade [29,30]. The main sugar used in many sports drinks is table sugar (i.e., sucrose), which is a disaccharide. Over time, sucrose (and other more complex sugars) can hydrolyze into their monosaccharide components [31] and increase the overall osmolality of the solution, a process that is exacerbated by storage in warm conditions [19,20]. Indeed, Gatorade demonstrated the highest absolute change in osmolality after 2 months in the heat (Δ 108 mmol/kg compared to baseline). Our results mirror what others have found in that osmolality may be strongly influenced by the quantity and quality of carbohydrates used (e.g., monosaccharides, disaccharides, polysaccharides, etc.) [29,32]. Other ingredients, such as artificial sweeteners and flavoring agents, may also be heat unstable. For example, the osmolality of Pedialyte Classic increased by 41 mmol/kg after 60 days in warm storage despite containing only dextrose (hydrolysis resistant). However, it also contained sucralose and acesulfame potassium, the effects of which are unknown with respect to osmolality. 

The beverage with the highest baseline osmolality was Speedlyte. Presently, a paucity of published data exists on this product. Speedlyte contains multiple forms of sugar and a unique electrolyte formulation where the electrolytes are encapsulated in liposomes (i.e., microelectrolytes technology™). Importantly, the manufacturers claim that the osmolality of this product is 188 mmol/kg [33]; however, over the course of this entire investigation, five separate bottles of this product were assayed and the osmolality values ranged from 406 to 410 mmol/kg. Moreover, this product demonstrated the second highest absolute changes in osmolality (Δ 90 mmol/kg increase compared to baseline) after 2 months at high temperatures. Although it is possible that the presence of liposomes (solids) in the sample might have spuriously elevated the osmolality measured by freezing point depression, the large sample volume (250 μL) should have minimized this potential [23,24]. For example, the water content of Speedlyte (97% water) was similar to the other commercial beverages tested (96–99% water); liposomes could have only contributed between 0.1% and 1.0% to the total [33]. In contrast, whole blood (~80% water), which contains considerable solids, can still have its osmolality accurately measured by freezing point depression when using a 250 μL volume [24]. In addition, similar outcomes were observed using a vapor pressure osmometer (data not shown). These results raise interesting and unresolved questions about liposome technology for use in ORS. 

The WHO currently recommends that a small amount of glucose should be added in an ORS to capitalize on sodium–glucose transporters that facilitate fluid movement [15]. enterade was the only product tested that does not contain glucose but instead contains electrolytes and amino acids to leverage amino acid coupled transporters that also facilitate fluid absorption [34]. It is interesting to note recent findings that small amounts of glucose (less than 1 millimolar) in a solution stimulates chloride secretion and as a consequence may reduce fluid absorption and may exacerbate diarrhea [35,36,37]. Furthermore, based upon the fact that the glucose-free product demonstrated the smallest change in osmolality over time, the degree to which glucose should be included in an ORS formulation may be questioned. The results of the current investigation have important implications for when these beverages are shipped and stored in locations where high ambient temperatures may be present, such as low-income countries with tropical climates [18,19]. 

The powdered versions of the ORS appeared to show better compliance with the recommended osmolality guidelines [15,16], but these powdered products can have their own limitations. For example, previous research has stressed that these products should be prepared extemporaneously to prevent alterations in product components over time [17]. However, if ORS needs to be used in areas without potable water, these beverages will not safely be reconstituted and will not be stable enough for storage. Lastly, some of the powdered ORS products contain components (e.g., rice powers) that have reduced solubility and thus alter the osmolality within these products (e.g., Dioralyte Relief) after reconstitution; thus, creating larger potential variation in measured osmolality values.

In summary, this investigation demonstrated that many commercially available pre-mixed ORS products currently do not adhere to recommended osmolality guidelines. Additionally, due to the high sugar content of some products used to treat diarrhea, their osmotic shelf-life stability may be questioned, especially when stored in the warm but ecologically valid environments. As the osmolality of ORS is important for optimal fluid absorption and patient acceptance (e.g., stomach upset), the findings of this study should be carefully considered in the design of future ORS products. 

## Appendix A

**Table A1:** Listing of ingredients for each premixed beverage tested.

Beverage Name	Ingredients
Pedialyte^®^ Classic mixed (fruit flavor)	Water, Dextrose. Less than 2% of: Citric Acid, Natural & Artificial Flavor, Potassium Citrate, Salt, Sodium Citrate, Sucralose, Acesulfame Potassium, Zinc Gluconate, and Yellow 6.
Pediatric Oral Electrolyte Solution (fruit flavor)	Water, Dextrose, Citric Acid, Potassium Citrate, Sodium Chloride, Sodium Citrate, Acesulfame Potassium, Zinc Gluconate, Natural & Artificial Fruit Flavor, Sucralose, FD&C Yellow #6.
Speedlyte^®^ (wild orange flavor)	Purified water; Less than 2% of: Dextrose, Sucrose, Liposomal Salt [Citrate, Chloride, Sodium, Potassium, Soy Lecithin, Xanthan Gum], Citric Acid, Sodium Benzoate, Stevia Extract, Monk Fruit, Beta Carotene, Rose Anthocyanin, Methylparaben, Sodium Metabisulfite, Natural Flavor.
enterade^®^AD ORS (orange flavor)	Water, Amino Acid Blend (L-Valine, L-Aspartic Acid, L-Serine, L-Isoleucine, L-Threonine, L-Lysine, L-Glycine, L-Tyrosine), sodium chloride, potassium citrate, trisodium citrate, natural flavor, magnesium citrate, calcium chloride, stevia.
Gatorade^®^ (orange flavor)	Water, Sugar, Dextrose, Citric Acid, Salt, Sodium Citrate, Monopotassium Phosphate, Gum Arabic, Natural Flavor, Sucrose Acetate Isobutyrate, Glycerol Ester of Rosin, Yellow 6
